# Effectiveness of the Non-restorative Cavity Control (NRCC) Approach in Managing Dentinal Caries in Primary Teeth: A Systematic Review

**DOI:** 10.7759/cureus.102458

**Published:** 2026-01-28

**Authors:** Mahdi M Alwusaybie, Guna Shekhar Madiraju, Hussain A Alhasan, Mohammed Afif Alshaks, Mohamad Hassan Alhafi, Ali Alkadem, Raed M Alnasser, Salman Alshamari, Mujtaba M Alhamoud, Ruqayah S Alhulaybi, Anwar N Almulhim

**Affiliations:** 1 Endodontics, Hail Dental Center, Ministry of Health, Hail, SAU; 2 Preventive Dental Sciences, King Faisal University, Hofuf, SAU; 3 Dentistry, Al-Ahsa Health Cluster, Ministry of Health, Al-Ahsa, SAU; 4 Dentistry, King Faisal University, Hofuf, SAU; 5 Dentistry, King Fahad Hospital, Hofuf, SAU

**Keywords:** dentinal caries, non-restorative cavity control, pediatrics, primary teeth, systematic review

## Abstract

Early childhood caries (ECC) is a highly prevalent, transmissible, and infectious disease affecting 60-90% of children worldwide. Conventional restorative therapy has traditionally been the mainstay of ECC management, but it often shows unfavorable outcomes in pediatric populations. Recently, there has been a shift toward minimally invasive interventions or non-restorative cavity control (NRCC). This review evaluated the effectiveness of NRCC in managing dentinal caries in primary teeth. A comprehensive search was conducted in Scopus, PubMed, MEDLINE, and Web of Science using keywords related to NRCC and primary dentition. Inclusion criteria were peer-reviewed randomized clinical trials (RCTs) published from 2015 to 2025, involving children ≤12 years with active dentinal lesions scored using the International Caries Detection and Assessment System (ICDAS) form 3 to 6. Studies were assessed for quality and risk of bias using the Cochrane Risk of Bias 2 (ROB-2) tool. Nine RCTs were finally included in the review. Most studies demonstrated that 38% silver diamine fluoride (SDF) effectively arrested dentinal caries, even with minimal application (30 seconds). One study reported that restorative techniques, such as the Hall technique, had higher survival rates than SDF. Nevertheless, SDF was well accepted by children and parents, who reported high satisfaction. Current evidence supports NRCC over conventional restorative therapy for managing dentinal caries in primary teeth. Despite aesthetic concerns, SDF remains an effective, safe, feasible, and well-accepted option for children and parents.

## Introduction and background

Dentinal caries is a chronic, transmissible, infectious disease affecting 60-90% of the general population, particularly children [1], and is a significant public health concern due to its considerable economic and health impact, especially in developing countries. Populations with low socioeconomic status are the most affected because of limited access to oral healthcare and insufficient awareness of oral care in children [2,3]. Caries develops from fermentable carbohydrate residues in the oral cavity, predominantly caused by the bacterium *Streptococcus mutans* (*S. mutans*), which produces organic acids [1]. These acids demineralize the tooth structure, leading to cavitation and lesion formation [4,5].

For decades, conventional restorative therapy has been the mainstay of early childhood caries (ECC) management. However, in pediatric populations, it has been associated with unfavorable outcomes such as high failure rates, high costs, and limited accessibility, particularly in underserved populations [6]. Such challenges can often lead to fear and poor cooperation among children during dental treatment, in addition to their innate fear of unfamiliar stimuli [7]. According to the ecological plaque hypothesis, ECC can be managed by focusing on oral hygiene, thereby disrupting the favorable environment for cariogenic bacteria [8]. A recent paradigm shift toward minimally invasive interventions for managing ECC has been observed. This approach is based on regular toothbrushing with fluoridated toothpaste, which removes the bacterial biofilm and reduces the progression of carious lesions. This promising strategy is referred to as non-restorative cavity control (NRCC) [9].

NRCC is known for its minimally invasive caries management approach for cavitated carious lesions that aims to arrest disease progression without the placement of a conventional restoration by integrating biofilm control as the primary therapeutic mechanism, selective modification of the cavity to ensure accessibility for effective plaque removal, adjunctive use of non-operative anticariogenic agents such as fluoride varnish or silver diamine fluoride (SDF) when indicated, behavioral and preventive interventions including oral hygiene and dietary counseling, and regular clinical monitoring rather than definitive restorative treatment [10,11]. Consistent with this preventive focus, studies have shown that fluoride varnish and SDF reduce ECC incidence by approximately 43% and 78%, respectively [12].

Therefore, NRCC provides an alternative to restorative or surgical interventions, overcoming challenges such as poor child cooperation, the need for anesthesia, anxiety, and pain [12]. The potential effectiveness of NRCC is supported by the American Dental Association recommendations that emphasize risk-based caries management, including non-restorative and minimally invasive preventive approaches, particularly for patients at moderate to high caries risk [13]. Additionally, organizations such as the European Federation of Conservative Dentistry, the European Organization for Caries Research, and the German Society for Conservative Dentistry recommend NRCC for the management of cavitated carious lesions [14]. Despite several recommendations, studies have reported relatively poor NRCC practice and implementation among dentists in different regions [15-17]. Moreover, there is a lack of evidence-based synthesis specifically focused on the use of NRCC for managing dentinal caries in primary teeth, particularly addressing outcomes such as caries arrest, progression, and complications or adverse events, as well as comparisons with restorative approaches. This knowledge gap highlights the need for a comprehensive overview of current evidence to guide clinicians, policymakers, and guideline developers. Accordingly, this review aimed to evaluate the effectiveness of NRCC in managing dentinal caries in primary teeth.

## Review

Methodology

Search Strategy

The study followed the Preferred Reporting Items for Systematic Reviews and Meta-Analyses (PRISMA) reporting guidelines [18]. The study's retrieval was from the Scopus, PubMed, MEDLINE, and Web of Science databases. The following keywords were used in the search to obtain the relevant articles: (("non-restorative cavity control" OR NRCC OR "nonrestorative" OR "non restorative" OR "cavity control" OR "non-operative caries" OR "non-invasive caries" OR "biological caries management")) AND (("primary teeth" OR "deciduous teeth" OR "primary dentition" OR child*)).

Eligibility Criteria

Inclusion and exclusion criteria:* *We included peer-reviewed English-language randomized clinical trials (RCTs), with or without a control group, published between 2015 and 2025, a period during which caries management has shifted toward more conservative, patient-centered strategies. There is now a stronger focus on biological methods that prioritize preservation over intervention, such as NRCC. This evolution has been guided by more nuanced caries risk assessment tools, clearer outcome measures, and internationally recognized guidelines that together encourage a minimally invasive philosophy in everyday practice. We included studies involving children aged 12 years or younger with active dentinal caries lesions on primary teeth, scored using the International Caries Detection and Assessment System (ICDAS) from 3 to 6 [19]. The studies had to manage caries using NRCC-like methods, including cleaning with sodium fluoride (NaF), SDF, stannous fluoride (SnF) toothpaste or gel, or chlorhexidine, and assess effectiveness in terms of caries arrest or progression as the primary outcome, with at least six months of follow-up. Secondary outcomes included adverse events, complications, and parents' satisfaction. Studies were excluded if they were observational, review articles, case reports/series, qualitative studies, in vitro studies, or protocols or if they involved adults or adolescents with enamel caries managed using restorative techniques.

Screening

All retrieved studies were independently evaluated by MMA, GSM, HAA, and MAA for eligibility. Duplicates were removed, followed by the screening of titles and abstracts. The four authors reviewed the remaining full-text articles to identify the relevant studies for inclusion in the systematic review. Discrepancies were resolved through discussion until consensus was reached.

Data Extraction and Synthesis

Data extraction was performed independently by two reviewers (MHA and AA) and cross-checked by another reviewer (RMN). The extracted data were organized into two tables. The first table included general study characteristics: last name of the first author, year, country, study design and duration, sample size (population or lesions), gender distribution, mean age, lesion type (cavitated or non-cavitated) and location, mean follow-up duration, and mean DMFT (decayed, missing, and filled teeth).

The second table focused on NRCC-related outcomes, including the following: intervention, control, type of outcome assessed, outcome value or effect size, adverse events or complications, and study conclusions. Any disagreements during data extraction were resolved through discussion among the reviewers (MHA, AA, and RMN) to ensure accuracy and consistency.

Quality Assessment

All relevant studies were assessed for quality and risk of bias using the Cochrane Risk of Bias 2 (ROB-2) tool for RCTs [20]. The assessment conducted by two reviewers (SAS and MMA) evaluated five main domains: bias arising from the randomization process, bias due to deviations from intended intervention, bias due to missing outcome data, bias in measurement of the outcome, and bias in selection of the reported result. Each domain was rated as low risk, some concerns, or high risk, and an overall risk of bias was assigned to each study. Any disagreements between reviewers were resolved through discussion with the third reviewer (RSA) to ensure consistency and accuracy.

Results

**Figure 1:**
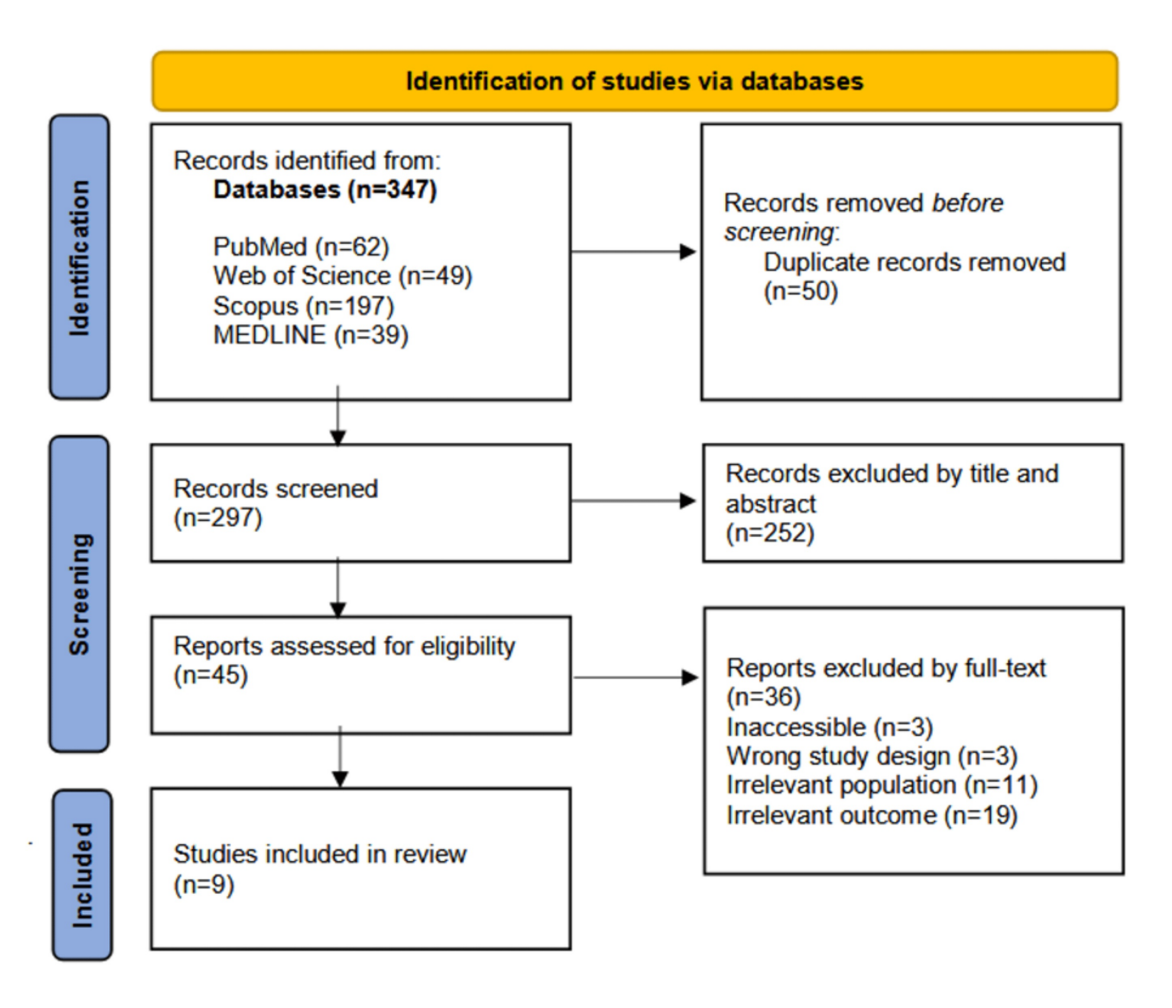
PRISMA flow diagram for the selection process PRISMA: Preferred Reporting Items for Systematic Reviews and Meta-Analyses

The systematic search initially yielded 347 articles. Of these, 50 duplicates were removed, followed by the exclusion of 252 articles based on title and abstract screening. An additional 36 articles were excluded after full-text review for failing to meet the eligibility criteria. Consequently, nine relevant studies were included in this review, as illustrated in Figure 1.

General Characteristics of the Included RCTs

As shown in Table 1, the included RCTs were published from 2020 to 2024 and varied in design, including prospective, cluster, open-label, parallel-group, patient-/parent-blinded, single-blinded or double-blinded, three-, four-, and six-arm, and multicenter RCTs. Four of the nine studies were conducted in India, and one each was conducted in the USA, the UK, Cambodia, Latvia, and Canada. A total of 5,277 children were recruited in the RCTs, aged from three to 11 years. Among them, 12,294 lesions were diagnosed, three studies highlighted cavitated lesions, and only one found both cavitated and non-cavitated lesions. The dentinal lesion's location ranged from posterior, anterior, occlusal, proximal, and smooth on primary molars. The minimum follow-up duration was six months, while the maximum was 12 months. Only four of nine studies reported the mean baseline DMFT index, which ranged from 0.7 to 6.8, suggesting variation in the baseline caries experience of the included population.

**Table 1:** General characteristics of the included RCTs RCTs: randomized clinical trials; P1: placebo four times per year; P2: placebo biannual; TF1: tiefenfluorid four times per year; TF2: tiefenfluorid biannual; SDF1: silver diamine fluoride four times per year; SDF2: silver diamine fluoride biannual; C+P: conventional + prevention; B+P: biologic and prevention; PA: prevention alone; ICDAS: International Caries Detection and Assessment System; IQR: interquartile range; DMFT: decayed, missing, and filled teeth

Author’s last name, year, country	Study design	Sample size (N)		Mean age (SD) (years)	Lesion type	Lesion location	Follow-up (months)	Mean (SD) baseline DMFT
		Subjects	Lesions					
Schroth et al., 2024, Canada [21]	Open-label, parallel-group RCT (Oct 2019 to Jun 2021)	84 (2 months: 28; 4 months: 28; 6 months: 28)	505	3.7 (1.2)	Cavitated (ICDAS=5-6)	Posterior: 205 (40.6%); anterior: 300 (59.4%)	2, 4, and 6	6.8 (4.5)
Maldupa et al., 2024, Latvia [22]	Six-arm, patient-/parent-blinded, superiority, placebo-controlled RCT	427 (P1: 70; P2: 72; TF1: 70; TF2: 72; SDF1: 71; SDF2: 72)	Mean: 7.28 (4.71)	3.8 (1.17)	NA	NA	12	6.58 (4.03)
Vaid et al., 2023, India [23]	Single-blinded RCT (Nov 2021 to 2022)	NA	50 (Grp 1: 27; Grp 2: 23)	3-5	Active (ICDAS=4-5)	Occlusal	6	NA
Ruff et al., 2023, the USA [24]	Single-blind, cluster, noninferiority RCT (Feb 2019 to Jun 2023)	2998 (1554 in experimental and 1444 control)	874	6.6 (1.2)	NA	NA	12	0.7 (1.4)
Undre et al., 2023, India [25]	RCT	120 (Grp 1: 40; Grp 2: 40; Grp 3: 40)	NA	5-8	Cavitated	Occlusal and proximal molars	3, 6, 9, and 12	NA
Thakur et al., 2022, India [26]	Prospective RCT	154 (Grp 1: 54; Grp 2: 49; Grp 3: 51)	154	7.55 (2.34)	Cavitated (ICDAS=5-6) and non-cavitated (ICDAS=3-4)	NA	0.75, 3, and 6	NA
Mani Prakash et al., 2022, India [27]	Double-blinded RCT	34	68 (Grp 1: 34; Grp 2: 34)	6-9	Cavitated (ICDAS=5)	Right and left molars	6 and 12	NA
Turton et al., 2021, Cambodia [28]	Four-armed, parallel-design cluster RCT	421 (Grp 1: 83 (19.7%); Grp 2: 148 (35.2%); Grp 3: 122 (29%); Grp 4: 68 (16.2%))	4496	7.6 (1.9), range 3-11	ICDAS=3-6	Occlusal: 639 (13.8%); proximal: 2844 (61.4%); smooth: 1146 (24.8%) on molars and incisors	6 and 12	NA
Innes et al., 2020, the UK [29]	Multicenter, three-arm, parallel-group RCT	1144 (C+P: 386; B+P: 381; PA: 377)	NA	3-7	NA	NA	Median: 33.8 m (IQR: 23.8-36.7)	2.7 (2.7)

Effectiveness of NRCC for Children's Primary Teeth Dentinal Caries Management

Table 2 summarizes the key findings regarding the effectiveness of the NRCC in managing dentinal caries in primary teeth across the included trials. Overall, seven of nine studies employed 38% SDF as an intervention, while one used it in the control group [27]. The SDF was applied either alone or in combination with 5% NaF [21,24], potassium iodide (KI) [28], and behavioral modifications [22]. In addition, placebo, high-viscosity glass ionomer cement/sealants, the Hall technique, and silver fluoride (AgF) were used as controls in the studies. 

**Table 2:** Effectiveness-related outcomes of NRCC interventions for managing dentinal caries in primary teeth in children *BM: behavioral modification, which included good oral hygiene and dietary practices. **Prevention included dietary investigation, toothbrushing, mouth rinsing, and topical fluoride varnish. SDF: silver diamine fluoride; SD: standard deviation; N: number; P1: placebo four times per year; P2: placebo biannual; TF1: tiefenfluorid four times per year; TF2: tiefenfluorid biannual; SDF1: silver diamine fluoride four times per year; SDF2: silver diamine fluoride biannual; NA: not available; ECC: early childhood caries; OR: odds ratio; GIS: glass ionomer sealant; GIC: glass ionomer cement; HT: Hall technique; CR: conventional restoration; NRCT: non-restorative caries treatment; NRCC: non-restorative cavity control; s: seconds; NaFV: sodium fluoride varnish; AgF: silver fluoride; KI: potassium iodide

Author's last name, year, country	NRCC intervention	Control	Primary outcome	Outcome measured/effect size	Secondary outcome (adverse event/complications)	Conclusion
Schroth et al., 2024, Canada [21]	38% SDF, along with 5% NaFV	NA	Caries arrest	Follow-up completion rates were 192/196 (98%) at 1 month, 159/166 (95.8%) at 4 months, and 103/143 (72%) at 6 months	NA	38% SDF and 5% NaFV at one- and four-month intervals were comparable and very effective in arresting ECC compared to six-month intervals
Maldupa et al., 2024, Latvia [22]	38% SDF, TF, with BM*	Placebo with BM	Reduction in major and minor complications	SDF2 was associated with a significantly lower risk of major complications (21.5%; OR=0.28; 95% CI: 0.11-0.72; p<0.05) compared with placebo	None	The SDF biannual application with BM effectively prevented major complications of ECC, along with high levels of children's and parents' satisfaction (96%)
Vaid et al., 2023, India [23]	38% SDF	High-viscosity GIC	% progression, lesion texture (hard), chairside time	SDF: 2 (7.4%); GIS: 9 (39.1%) (p=0.017). SDF: 19 (70.4%) (p<0.05). SDF: 4.28 min; GIS: 7.66 min (p=0.039)	NA	38% SDF and high-viscosity GIC for the occlusal surface cavity appear to be effective in arresting the progress of dentinal caries in primary teeth
Ruff et al., 2023, the USA [24]	38% SDF with 5% fluoride varnish	GIS and atraumatic restoration with fluoride	Caries arrest, absence of new incidence	Grp 1: 0.56 (0.04); Grp 2: 0.46 (0.04) (difference: −0.11; 95% CI: −0.22 to 0.01) (OR: 1.49; 95% CI: 0.91-2.44; p=0.1). Grp 1: 0.81 (0.02); Grp 2: 0.82 (0.02) (difference: 0.01; 95% CI: −0.04 to 0.06) (OR: 0.93; 95% CI: 0.68-1.27; p=0.62)	None	SDF with fluoride varnish was noninferior to GIS and atraumatic restorations with fluoride varnish for caries arrest and prevention
Undre et al., 2023, India [25]	38% SDF	HT (stainless steel crown with GIC) and CR	Survival rate	HT: 37 (92.5%); SDF: 34 (85%); CR: 28 (70%); p=0.026 (SDF vs HT); p=0.009 (CR vs HT); p=0.006 (SDF vs CR)	NA	HT performed better than CR. NRCT was more acceptable to patients than CR
Thakur et al., 2022, India [26]	38% SDF applied for 30, 60, and 120 s	NA	Caries arrest	At 6 months: 30 s: 51 (33.12%); 60 s: 49 (31.81%); 120 s: 50 (32.47%)	NA	SDF is effective in controlling caries progression in both cavitated and non-cavitated lesions with a minimal time duration of application (30 s)
Mani Prakash et al., 2022, India [27]	2-5% NaF varnish	1-38% SDF	Caries arrest	At 6 months: SDF: 82%; NaF: 45% (p=0.002). At 12 months: SDF: 77%; NaF: 42% (p=0.004)	NA	SDF was more effective in arresting dentinal caries in primary molars compared to 5% NaF varnish
Turton et al., 2021, Cambodia [28]	SDF with/without KI	AgF with/without KI	Caries arrest rate	Grp 1 (SDF): 77.3%. Grp 2 (SDF+KI): 65.4%. Grp 3 (AgF): 75.3%. Grp 4 (AgF+KI): 51.2%. KI arrest (12 m; OR: 0.25; 95% CI: 0.19, 0.34; p<0.001). KI discoloration avoidance (12 m; OR: 6.08; 2.36, 15.67)	At baseline: only transient gingival irritation. Pulpal involvement at 12 m; SDF: 62 (13.6%); AgF: 84 (10.6%); +KI: (OR: 2.21; 95% CI: 1.33-3.68; p=0.002)	AgF and SDF can effectively arrest carious lesions on primary teeth. The use of KI is associated with poorer caries control but better aesthetic outcomes
Innes et al., 2020, the UK [29]	Prevention alone (PA)**, conventional + prevention (C+P), and biologic and prevention (B+P)	NA	Dental pain/infection	C+P: 42% (148 of 352). B+P: 40% (141 of 352). PA: 45% (161 of 354)	NA	No significant difference among the three approaches for incidence or number of episodes of dental pain and/or infection

The primary outcome was assessed using the ICDAS criteria in all trials to facilitate a valid, standardized comparison of the findings. Moreover, the caries arrest rate (CAR) showed that 38% SDF, used either alone or combined with 5% NaF, applied for a minimal time (30 seconds), was significantly effective in arresting cavitated and non-cavitated active dentinal caries lesions in children compared to the controls. Moreover, SDF was significantly associated with a lower progression rate than the high-viscosity GIS (7.4% vs 39.1%; p=0.017).

Likewise, AgF demonstrated notable efficacy in managing ECC on primary teeth. Furthermore, in terms of reducing minor complications (OR=0.28; 95% CI: 0.11, 0.72; p<0.05) and major complications (OR=0.16; 95% CI: 0.05, 0.50; p=0.002), respectively, the biannual SDF had a significantly lower rate (21.5%) compared to placebo. Additionally, Turton et al. emphasized the significant role of KI in reducing the SDF-induced discoloration (OR: 6.08; 95% CI: 2.36-15.67) and limiting the progression of the dentinal caries (OR: 0.25; 95% CI: 0.19-0.34; p<0.001), respectively, at 12 months [28]. Parents' satisfaction was assessed in one study, which reported that 96% of parents provided positive feedback on the biannual application of SDF, along with behavioral modifications. The adverse event reported in one study was transient gingival irritation following the application of 38% SDF with KI. Also, pulpal involvement was observed at 12 months (OR: 2.21; 95% CI: 1.33-3.68; p=0.002).

**Figure 2:**
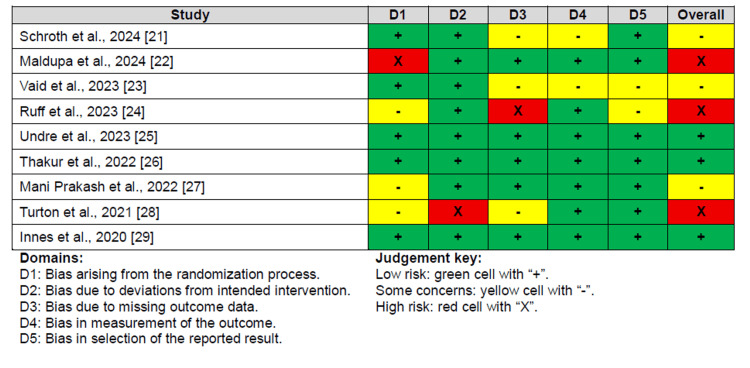
Risk of bias assessment for the included RCTs using the ROB-2 tool RCTs: randomized clinical trials; ROB-2: Risk of Bias 2

The risk of bias assessment revealed that three studies had a high risk [22,24,28], three studies had some concerns [21,23,27], and three studies had a low risk [25,26,29]. The primary issues in most RCTs were bias resulting from the randomization process, missing outcome data, inadequate outcome measurement, and selection bias in the reported results, as illustrated in Figure 2.

Discussion

This systematic review evaluated the effectiveness of NRCC in managing dentinal caries in primary teeth. Among the nine included RCTs, seven studies investigated 38% SDF as the intervention, one used 38% SDF as the control, and one compared three modalities: preventive, conventional, and biologic restorative techniques. All studies assessing 38% SDF reported its significant effectiveness in arresting dentinal caries in children, even with minimal application time (30 seconds) [26]. Only one study found that restorative methods, such as the Hall technique, demonstrated a higher survival rate than SDF; nevertheless, SDF was well accepted by both children and parents, who expressed satisfaction with its use.

Favorable outcomes with SDF were observed whether it was used alone or in combination with behavioral modifications, 5% NaF, or KI. In 2014, the US Food and Drug Administration approved SDF for off-label use to arrest caries in both dentinal and enamel lesions in children. Its increasing recognition as an NRCC option in primary teeth is attributed to its cost-effectiveness, safety, minimal chair time, non-invasiveness, and lack of aerosol generation [30]. The caries-arresting mechanism of SDF primarily relies on silver's antibacterial properties and the formation of a sclerotic layer on the dentinal surface, which promotes the remineralization of enamel and dentin [31].

Consequently, the American Dental Association clinical practice guideline recommends a biannual application of 38% SDF for arresting advanced cavitated caries in primary teeth [32]; however, Maldupa et al. and Gao et al. reported that biannual SDF applications were more effective in preventing major complications and achieving higher success rates in managing ECC [22,33].

Our review confirms earlier observations by Cabalén et al., who reported that SDF achieved significant CAR of 37.5-64.1% at 12 months, 42-57% at 24 months, and 48% at 30 months [34]. Additionally, a systematic review and meta-analysis by Urquhart et al. provided evidence on NRCC management of caries, demonstrating that the biannual application of 38% SDF on primary teeth resulted in better dental outcomes compared to 12% SDF (RR: 1.29; 95% CI: 1.21-1.38) and annual 38% SDF (RR: 1.13; 95% CI: 1.07-1.20) [35]. Another systematic review by Contreras et al. concluded that 30-38% SDF solutions were more effective than 5% NaF varnish in arresting dentinal caries in primary dentition [36]. In contrast, Lenzi et al. reported that 5% NaF varnish was effective in managing caries lesions in both primary and permanent teeth when compared with 1.23% acidulated phosphate fluoride (APF) gel or no treatment [37].

Furthermore, an umbrella review found that SDF had 90% effectiveness in arresting the progression of caries lesions in children after two years of follow-up, compared with other active treatments [38]. However, Zhi et al. noted that the effectiveness of SDF in caries arrest was primarily limited to occlusal and smooth surfaces, while buccal and lingual surfaces were less responsive to SDF in the included trials [39]. SDF is considered safe, with no major adverse effects reported in the literature. However, tooth dark discoloration remains the primary concern associated with SDF use, unlike with 5% NaF [40]. Seifo et al. reported that some children experienced school bullying related to the black staining of their teeth [41]. Despite this drawback, SDF remains a favorable NRCC option for both parents and children [42].

Strengths and Limitations

This review included only RCTs, which represent the highest level of evidence for assessing the effectiveness of NRCC. In addition, the inclusion of studies with long-term follow-up periods strengthens the findings, as such durations are often not feasible in retrospective designs. Furthermore, the exclusive use of the ICDAS as an eligibility criterion ensured a reproducible and validated comparison across trials. Restricting inclusion to dentinal lesions also aligned with the review's primary objective.

Nevertheless, several limitations may affect the robustness of the findings. First, the relatively small number of included studies limits the generalizability of the results. Second, most studies focused primarily on SDF, thereby providing limited evidence on other NRCC interventions, such as resin-based techniques and APF. Additionally, this review did not provide sufficient information on the influence of parental adherence to behavioral modifications or oral hygiene practices on the quality of life of affected children. Finally, the review protocol was not prospectively registered in a public repository such as the International Prospective Register of Systematic Reviews (PROSPERO). While the methodology was rigorously planned and adhered to PRISMA guidelines, the absence of prior registration should be considered when interpreting the findings.

Recommendations

Future research should focus on conducting high-quality clinical trials evaluating NRCC techniques beyond SDF, including casein phosphopeptide-amorphous calcium phosphate (CPP-ACP), chlorhexidine, and other fluoride-containing materials. Greater emphasis should also be placed on assessing parents' and children's experiences, acceptability, and perceptions of NRCC techniques to enhance compliance and long-term success.

In addition, the implementation of educational and training programs for dentists and pediatricians is recommended to increase the awareness of the importance of NRCC and reduce the unnecessary use of restorative techniques. Clinicians should also encourage patients and caregivers to adhere to routine oral hygiene practices and regular dental care, which are essential components of effective caries management.

## Conclusions

The current evidence favors NRCC over conventional restorative management for dentinal caries in primary teeth. Despite aesthetic concerns, SDF remains an effective, safe, feasible, and satisfactory option from both parents' and children's perspectives. In addition, 5% NaF, KI, and promoting oral hygiene practices contribute significantly to the management of ECC. Notably, NRCC offers a suitable alternative when conventional restorative approaches are limited by cost, poor patient cooperation, or clinical feasibility. Nevertheless, additional high-quality clinical trials are needed to improve patient-reported outcomes and support the development of evidence-based clinical guidelines.
